# Supplementary material for: Gastroesophageal reflux symptoms and sleep quality among medical students at a private university in Lima, Peru: A cross-sectional study
Source: PLoS One. 2026 Jun 1;21(6):e0348891. doi: 10.1371/journal.pone.0348891 (PMC13225408; doi:10.1371/journal.pone.0348891)
Supplement: S1 Table — (DOCX) [file pone.0348891.s001.docx]

S1 Table. Frequency of GERD symptoms (n = 171).

| Symptom | Never n (%) | Rarely n (%) | Sometimes n (%) | Frequently n (%) | Always n (%) |
| --- | --- | --- | --- | --- | --- |
| Sensation of stomach acidity | 33 (19.3) | 69 (40.4) | 53 (31.0) | 15 (8.8) | 1 (0.6) |
| Sensation of stomach bloating | 24 (14.0) | 53 (31.0) | 59 (34.5) | 27 (15.8) | 8 (4.7) |
| Sensation of stomach heaviness after eating | 12 (7.0) | 46 (26.9) | 67 (39.2) | 39 (22.8) | 7 (4.1) |
| Rubs the chest with the hand (subconsciously) | 65 (38.0) | 53 (31.0) | 36 (21.1) | 12 (7.0) | 5 (2.9) |
| Feels ill after eating | 74 (43.3) | 52 (30.4) | 31 (18.1) | 12 (7.0) | 2 (1.2) |
| Heartburn after meals | 62 (36.3) | 57 (33.3) | 33 (19.3) | 17 (9.9) | 2 (1.2) |
| Burning sensation in the throat | 81 (47.4) | 61 (35.7) | 21 (12.3) | 6 (3.5) | 2 (1.2) |
| Feeling of fullness while eating | 29 (17.0) | 57 (33.3) | 60 (35.1) | 23 (13.5) | 2 (1.2) |
| Food sticking while swallowing | 114 (66.7) | 36 (21.1) | 15 (8.8) | 5 (2.9) | 1 (0.6) |
| Acid or bitter liquid rising to the throat | 67 (39.2) | 61 (35.7) | 34 (19.9) | 6 (3.5) | 3 (1.8) |
| Frequent belching | 42 (24.6) | 76 (44.4) | 27 (15.8) | 23 (13.5) | 3 (1.8) |
| Heartburn when bending over | 85 (49.7) | 46 (26.9) | 34 (19.9) | 4 (2.3) | 2 (1.2) |
