# Supplementary material for: Gastroesophageal reflux symptoms and sleep quality among medical students at a private university in Lima, Peru: A cross-sectional study
Source: PLoS One. 2026 Jun 1;21(6):e0348891. doi: 10.1371/journal.pone.0348891 (PMC13225408; doi:10.1371/journal.pone.0348891)
Supplement: S2 Table — (DOCX) [file pone.0348891.s002.docx]

S2 Table. Sleep quality according to the components of the Pittsburgh Sleep Quality Index (PSQI) (n = 171).

| PSQI component | Mean ± SD |
| --- | --- |
| Component 1. Subjective sleep quality | 1.04 ± 0.96 |
| Component 2. Sleep latency | 1.10 ± 0.87 |
| Component 3. Sleep duration | 1.73 ± 0.94 |
| Component 4. Habitual sleep efficiency | 0.80 ± 1.10 |
| Component 5. Sleep disturbances | 1.20 ± 0.58 |
| Component 6. Use of sleep medication | 0.41 ± 0.71 |
| Component 7. Daytime dysfunction | 1.54 ± 0.91 |
| Total PSQI score | 7.82 ± 3.26 |
