# Supplementary material for: Gastroesophageal reflux symptoms and sleep quality among medical students at a private university in Lima, Peru: A cross-sectional study
Source: PLoS One. 2026 Jun 1;21(6):e0348891. doi: 10.1371/journal.pone.0348891 (PMC13225408; doi:10.1371/journal.pone.0348891)
Supplement: Attachment — Submitted filename: Sleep quality and GERD 1.docx [file pone.0348891.s004.docx]

Sleep quality and GERD

**Overall Assessment**

This manuscript addresses an important and relevant topic, the association between GERD symptoms and sleep quality among medical students. The study is generally well structured, uses validated instruments, and applies appropriate statistical methods. The results are clearly presented and the discussion demonstrates good engagement with existing literature. However, there are several important issues across sections, particularly related to clarity of the knowledge gap, methodological transparency, handling of confounding, statistical reporting, and interpretation of findings, which should be addressed to improve scientific rigor and publication readiness. Below, specific examples from the manuscript are highlighted to guide revision.

**Title**

The title is clear but slightly redundant. For example, the phrasing:

*“Association between gastroesophageal reflux symptoms and sleep quality…: a cross-sectional study”*

uses both “association between” and “cross-sectional study,” which both imply non-causal relationships. While acceptable, this creates unnecessary wordiness. Consider simplifying to:

*“Gastroesophageal reflux symptoms and sleep quality among medical students…”*

**Keywords**

The choice of keywords should prioritize **content and indexing value rather than methodological descriptors**. Inclusion of the study design (e.g., “cross-sectional study”) is generally unnecessary unless explicitly required by the journal. Keywords should instead emphasize the population (medical students), condition (GERD), outcome (sleep quality), and context (Peru or university setting). Refining the keywords in this way would improve discoverability and alignment with indexing standards.

**Abstract**

The abstract presents the main components of the study; however, it would benefit from clearer articulation of the knowledge gap and study contribution.

For example, while results are presented, it is not clear:

- What was unknown before this study
- How this study advances knowledge

The authors should explicitly state:

What is unknown in this specific population or region i.e what gap in Latin American or Peruvian student populations this study addresses.

A clear statement such as:

“However, evidence on this association among medical students in Latin America, particularly Peru, remains limited.”

**Methods**

The methods section is generally well described, with clear reporting of study design, ethical approval, informed consent procedures, and data collection using an online questionnaire. The use of validated tools (PSQI and FSSG) is a major strength, and the statistical approach using Poisson regression with robust variance is appropriate.

However, several important limitations remain. The description of the study setting is insufficient and requires more contextual detail about the institution and study population. The manuscript states:*“a private university (Universidad Peruana Unión) in Lima, Peru”*. However, there is no further descriptions such as type of institution structure of the medical program, student distribution. This detail to would improve interpretability and generalizability.

The sample size calculation lacks transparency, as key parameters such as allowable error (e.g., 0.05), formula used, and adjustment for non-response, are not reported and whether adjusting for a finite population was done, limiting reproducibility. The use of non-probabilistic convenience sampling introduces potential selection bias, which should be more explicitly acknowledged.

Importantly, confounder selection is incomplete, with omission of key variables such as mental health factors (stress, anxiety, depression) and medications affecting sleep, both of which are strongly associated with GERD and sleep quality. This introduces a high risk of residual confounding.

Additionally, there is no description of data quality control procedures (e.g., duplicate checks, completeness verification), which is particularly important for online surveys. The statement that age was categorized to address multicollinearity reflects a misunderstanding of the concept and should be revised.

**Results**

The results are logically organized and clearly presented, moving from participant flow to descriptive statistics, bivariate analysis, and multivariable analysis. The use of prevalence ratios with confidence intervals is appropriate and strengthens the analysis.

However, there is over-reliance on p-values, particularly in the bivariate analysis, without reporting effect sizes. The manuscript states: *“prevalence was significantly higher among students who reported NSAID consumption (93.5%, p=0.016)”* This reports significance but not effect size. It might be helpfull to include a table a table with effect size and their direction i.e (cPR) with 95% CI

Table 2 presents only frequencies and p-values, without the magnitude, which limits interpretation. Additionally, chi-square and related tests only demonstrate the presence of association, not its magnitude or direction, and this limitation is not acknowledged. Also, the legend states:*“p value calculated using Chi-square test, Fisher’s exact test, or Mann–Whitney U test”* However, it does not specify which test applies to which variable, reducing clarity

Some findings are described in a way that may imply interpretation rather than neutral reporting. The use of both continuous and categorical GERD variables is not clearly justified, which may lead to inconsistent interpretation. Table formatting also requires improvement, including clearer separation of counts and percentages for table 1

Furthermore, there is no mention of missing data or how it was handled. The very high prevalence estimates (GERD and poor sleep quality) are presented without sufficient contextual caution.

**Discussion**

The discussion is well structured and provides a clear summary of findings, with strong comparison to existing literature. The contextualization within Peruvian and global studies is appropriate and well supported. However they do not explain these results in their context .i.e why prevalence is high in medical students at this university

Additionally, the discussion does not clearly articulate **what this study adds to existing knowledge**, which weakens its scientific contribution. There is also some **overinterpretation of findings**, with language suggesting causal mechanisms that are not supported by the cross-sectional design. Additionally, the discussion implies independent associations for some variables that were not fully assessed in multivariable analyses. The interpretation of the dose–response relationship is slightly overstated and should be presented more cautiously.

The section combining “implications and recommendations” lacks clear separation between interpretation and actionable guidance. Furthermore, some recommendations extend beyond the study findings and are not fully supported by the data.

The omission of key confounders, particularly mental health variables, is acknowledged but not sufficiently emphasized in terms of its impact on results.

**Their limitations are missing the following aspects**

- **Selection bias insufficiently specified.** The authors state:*“Convenience sampling may limit external validity and generate selection bias.”* While correct, this is too generic and does not reflect the **actual recruitment process** described in the methods (i.e., recruitment during an academic/health-related event and via WhatsApp). This approach likely selected students who were: more **engaged**, more **health-conscious** and more likely to report or recognize symptoms. This may bias both and poor sleep and GERD prevalence. Therefore limitations should explicitly state that recruitment from an academic/health-related gathering and online dissemination may have preferentially included more health-aware participants, introducing selection bias.
- **Online survey limitations not acknowledged. The authors** mentions reporting bias but does not explicitly acknowledge that data were collected via an online, self-administered questionnaire, that might have been prone to self-report bias, recall bias, lack of supervision, with potential duplicate or careless responses. Authors should add a specific limitation related to the use of an online self-administered survey and its implications for data quality.
- **Residual confounding underemphasized.** The authors state:*“variables such as academic stress or other mental health variables were not measured…”* This is appropriate but **understated.** Mental health factors (stress, anxiety, depression) are strongly associated with both GERD and sleep quality. Their omission is a **major threat to internal validity. t**his statement should emphasize that omission of these variables may have significantly biased the observed associations.
- **Omission of medications affecting sleep as a variable. Authors dont** mention use of sleep medications, antidepressants or stimulants as important confounder affecting sleep quality only NSAIDs were assessed. They should acknowledge omission of medications affecting sleep as an additional source of residual confounding.
- **Reporting bias explanation could be strengthened.** The authors state: *“students experiencing these problems may have been more likely to respond…”*This is valid but could be more clearly linked to voluntary participation, online format, and recruitment channels
- **Lack of local validation of measurement tools.** The authors state:*“validated and widely used instruments were employed.* While PSQI has been validated in Peruvian university students. The **FSSG tool** was originally developed in a different population (Japan). Local validation in Peruvian students is **not clearly established**, with reliance on a student thesis. Therefore, the statement that both tools are “validated” may be **partially overstated**

The strengths section is relatively brief and could be expanded to include i.e appropriate analytical approach (use of prevalence ratios) and use of standardized instruments in a local context

**Conclusion and Recommendations**

The conclusion is well written and aligned with the study aims and findings. It appropriately summarizes the association between GERD and poor sleep quality without making causal claims.

However, recommendations are not clearly integrated with the conclusion and are not consistently derived from the study findings. There should be a clearer linkage between the results and the recommendations provided. Additionally, recommendations should be presented alongside the conclusion in a concise and evidence-aligned manner, ensuring they do not extend beyond what the data support.

**Critique of Conclusion and Recommendations**

**Overall Assessment**

The conclusion is **clear, well-structured, and aligned with the study aims**, appropriately summarizing, high prevalence of GERD and poor sleep quality, the observed association and the dose–response relationship. However, the **recommendations are vague, not sufficiently actionable, and not tightly linked to the study variables and findings**. Forexample, the statement:*“implementing systematic screening… and developing timely interventions…”*is **broad and non-specific**. It does not indicate w**hat type of screening, how should it be implemented nor what interventions specifically should be implemented.** This limits usefulness for policy, clinical practice and University health programs

Additionally there is n**o clear link between GERD management and sleep improvement**. The conclusion identifies GERD as a key factor but does not answer the question’**What should be done about GERD to improve sleep quality in terms of lifestyl** interventions, clinical management strategies, and behavioral modifications

**Besides there is Overgeneralization of “screening”** The recommendation:*“systematic screening for both conditions”* Screening is suggested without justification of feasibility and indication of where/how (e.g., student clinics

**Final Recommendation**

This manuscript addresses a relevant and important topic and demonstrates several methodological strengths, including the use of validated tools and appropriate analytical techniques. However, revisions are required to improve **clarity, methodological transparency, handling of confounding, statistical reporting, and interpretation of findings**. Addressing these issues will significantly strengthen the manuscript and enhance its suitability for publication.

Here is your **revised, detailed peer reviewer report with specific, text-linked examples**, so authors can clearly identify exactly what to revise. All comments are grounded in the manuscript content you provided .

**Overall Assessment**

This manuscript addresses an important topic—the association between GERD symptoms and sleep quality among medical students. The study is generally well organized and uses appropriate tools and analysis. However, several issues related to **clarity, methodological transparency, statistical reporting, and interpretation** need to be addressed. Below, specific examples from the manuscript are highlighted to guide revision.

**Title**

The title is clear but slightly redundant. For example, the phrasing:

*“Association between gastroesophageal reflux symptoms and sleep quality…: a cross-sectional study”*

uses both “association between” and “cross-sectional study,” which both imply non-causal relationships. While acceptable, this creates unnecessary wordiness. Consider simplifying to:

*“Gastroesophageal reflux symptoms and sleep quality among medical students…”*

**Keywords**

Keywords should focus on **content rather than study design**. If “cross-sectional study” is included, it adds limited indexing value since the design is already stated in the title and methods. Instead, prioritize terms such as:

- GERD
- Sleep quality
- Medical students
- Peru

**Abstract**

The abstract reports results but does not clearly state the **knowledge gap or contribution**. For example, while results are presented, it is not clear:

- What was unknown before this study
- How this study advances knowledge

The authors should explicitly state:

what gap in Latin American or Peruvian student populations this study addresses.

**Methods**

**Study Setting**

The manuscript states:

*“a private university (Universidad Peruana Unión) in Lima, Peru”*

However, there is no further description of:

- Type of institution
- Structure of the medical program
- Student distribution

👉 Add contextual detail to improve interpretability and generalizability.

**Sample Size Calculation**

The manuscript reports:

*“resulting in a minimum required sample size of 160 participants”*

However, it does not specify:

- Allowable error (e.g., 0.05)
- Formula used
- Adjustment for non-response

👉 This makes the calculation **non-reproducible**. Add these details explicitly.

**Confounders (Critical Issue)**

The manuscript includes variables such as:

NSAIDs, energy drinks, coffee, alcohol, tobacco

However, it omits key confounders such as:

- **Stress**
- **Anxiety**
- **Depression**
- **Medications affecting sleep (e.g., sedatives, antidepressants)**

This is particularly important given statements in the discussion such as:

*“high prevalence of depression and anxiety”*

👉 These variables were acknowledged conceptually but **not measured or adjusted for**, introducing **residual confounding**.

**Multicollinearity**

The manuscript states:

*“age was categorized into tertiles to avoid this issue”*

👉 This is incorrect. Categorizing age does **not resolve multicollinearity**, which occurs between variables, not within a single variable. This requires correction.

**Data Quality Control**

No description is provided on:

- Duplicate response prevention
- Missing data handling
- Logical checks

Given the use of:

*“Microsoft Forms” and WhatsApp distribution*

👉 Data quality assurance is essential and should be described.

**Results**

**Over-reliance on p-values**

The manuscript states: *“prevalence was significantly higher among students who reported NSAID consumption (93.5%, p=0.016)”*

👉 This reports significance but **not effect size**.

✔ Recommendation:

- Include **crude prevalence ratios (cPR) with 95% CI in Table 2**

**Table 2 Issues**

Table 2 presents:

- Frequencies
- p-values

But does **not include effect estimates**

👉 This limits interpretation. Also:

- The legend states:

*“p value calculated using Chi-square test, Fisher’s exact test, or Mann–Whitney U test”*

However, it does not specify **which test applies to which variable**, reducing clarity.

**Interpretation within Results**

The statement:

*“a linear and directly proportional relationship was observed…”*

👉 This is **interpretative language**, not purely descriptive.

✔ Replace with:

“a positive association was observed…”

**High Prevalence Values**

The manuscript reports:

- GERD: **76.6%**
- Poor sleep: **84.8%**

👉 These are unusually high but not critically discussed in results.

**Discussion**

**Lack of Explicit Contribution**

The manuscript compares with literature but does not clearly state:

- What is new

For example:

Multiple comparisons are provided, but no statement such as
“This study adds…” or “This study is the first to…”

👉 Add a clear contribution statement.

**Overinterpretation**

The manuscript states:

*“could be explained by… unhealthy dietary and behavioral habits”*

👉 This implies causality, which is not supported by the cross-sectional design.

**Independent Associations (Incorrect Implication)**

The manuscript discusses factors such as:

- Diet
- NSAIDs
- Energy drinks

👉 However, these were not analyzed as **independent predictors in separate models**, so they should not be presented as independently associated.

**Implications vs Recommendations**

The section:

*“Implications and recommendations”*

mixes:

- Interpretation
- Actionable recommendations

👉 These should be **separated clearly**

**Recommendations Not Fully Supported**

Recommendations include:

*“pharmacological treatment… comprehensive mental health approaches”*

👉 These extend beyond:

- Variables measured in the study

**Residual Confounding Underemphasized**

The manuscript states:

*“variables such as academic stress or other mental health variables were not measured”*

👉 This is a **major limitation**, but it is not strongly emphasized in the discussion.

**Dose–Response Overstatement**

The manuscript states:

*“strengthens the plausibility of the association”*

👉 In cross-sectional studies, this should be phrased more cautiously.

**Conclusion and Recommendations**

The conclusion is well aligned with the results and appropriately avoids causal language. However:

- Recommendations are not clearly linked to findings
- They are not presented alongside the conclusion in a structured way

👉 Ensure:

- Recommendations are **directly derived from findings**
- They are clearly integrated with the conclusion

**🔑 Final Recommendation**

The manuscript addresses an important topic and demonstrates several strengths, including appropriate tools and analysis. However, revisions are required to improve:

- **Clarity of contribution to knowledge**
- **Handling of confounding (especially mental health and medications)**
- **Statistical reporting (effect sizes in Table 2)**
- **Separation of results vs interpretation**
- **Alignment between findings and recommendations**

Addressing these issues will substantially strengthen the manuscript and improve its suitability for publication.

**Final Recommendation**

This manuscript addresses a relevant and important topic and demonstrates several methodological strengths, including the use of validated tools and appropriate analytical techniques. However, revisions are required to improve **clarity, methodological transparency, handling of confounding, statistical reporting, and interpretation of findings**. Addressing these issues will significantly strengthen the manuscript and enhance its suitability for publication.

If you want, I can next:

- Convert this into a **journal submission form (with rating scores + recommendation: Major revision)**
- Or **rewrite weak sections (methods/discussion) directly to publication standard**

rewrite this providing specific examples from the manuscript as describedin pasted text some comments are general and authors may not pinpoint the refered text in their manuscript

Here is your **revised, detailed peer reviewer report with specific, text-linked examples**, so authors can clearly identify exactly what to revise. All comments are grounded in the manuscript content you provided .

**🧾 Peer Reviewer Report (With Specific, Text-Linked Comments)**

**Overall Assessment**

This manuscript addresses an important topic—the association between GERD symptoms and sleep quality among medical students. The study is generally well organized and uses appropriate tools and analysis. However, several issues related to **clarity, methodological transparency, statistical reporting, and interpretation** need to be addressed. Below, specific examples from the manuscript are highlighted to guide revision.

**Title**

The title is clear but slightly redundant. For example, the phrasing:

*“Association between gastroesophageal reflux symptoms and sleep quality…: a cross-sectional study”*

uses both “association between” and “cross-sectional study,” which both imply non-causal relationships. While acceptable, this creates unnecessary wordiness. Consider simplifying to:

*“Gastroesophageal reflux symptoms and sleep quality among medical students…”*

**Keywords**

Keywords should focus on **content rather than study design**. If “cross-sectional study” is included, it adds limited indexing value since the design is already stated in the title and methods. Instead, prioritize terms such as:

- GERD
- Sleep quality
- Medical students
- Peru

**Abstract**

The abstract reports results but does not clearly state the **knowledge gap or contribution**. For example, while results are presented, it is not clear:

- What was unknown before this study
- How this study advances knowledge

The authors should explicitly state:

what gap in Latin American or Peruvian student populations this study addresses.

**Methods**

**Study Setting**

The manuscript states:

*“a private university (Universidad Peruana Unión) in Lima, Peru”*

However, there is no further description of:

- Type of institution
- Structure of the medical program
- Student distribution

👉 Add contextual detail to improve interpretability and generalizability.

**Sample Size Calculation**

The manuscript reports:

*“resulting in a minimum required sample size of 160 participants”*

However, it does not specify:

- Allowable error (e.g., 0.05)
- Formula used
- Adjustment for non-response

👉 This makes the calculation **non-reproducible**. Add these details explicitly.

**Confounders (Critical Issue)**

The manuscript includes variables such as:

NSAIDs, energy drinks, coffee, alcohol, tobacco

However, it omits key confounders such as:

- **Stress**
- **Anxiety**
- **Depression**
- **Medications affecting sleep (e.g., sedatives, antidepressants)**

This is particularly important given statements in the discussion such as:

*“high prevalence of depression and anxiety”*

👉 These variables were acknowledged conceptually but **not measured or adjusted for**, introducing **residual confounding**.

**Multicollinearity**

The manuscript states:

*“age was categorized into tertiles to avoid this issue”*

👉 This is incorrect. Categorizing age does **not resolve multicollinearity**, which occurs between variables, not within a single variable. This requires correction.

**Data Quality Control**

No description is provided on:

- Duplicate response prevention
- Missing data handling
- Logical checks

Given the use of:

*“Microsoft Forms” and WhatsApp distribution*

👉 Data quality assurance is essential and should be described.

**Results**

**Over-reliance on p-values**

The manuscript states:

*“prevalence was significantly higher among students who reported NSAID consumption (93.5%, p=0.016)”*

👉 This reports significance but **not effect size**.

✔ Recommendation:

- Include **crude prevalence ratios (cPR) with 95% CI in Table 2**

**Table 2 Issues**

Table 2 presents:

- Frequencies
- p-values

But does **not include effect estimates**

👉 This limits interpretation. Also:

- The legend states:

*“p value calculated using Chi-square test, Fisher’s exact test, or Mann–Whitney U test”*

However, it does not specify **which test applies to which variable**, reducing clarity.

**Interpretation within Results**

The statement:

*“a linear and directly proportional relationship was observed…”*

👉 This is **interpretative language**, not purely descriptive.

✔ Replace with:

“a positive association was observed…”

**High Prevalence Values**

The manuscript reports:

- GERD: **76.6%**
- Poor sleep: **84.8%**

👉 These are unusually high but not critically discussed in results.

**Discussion**

**Lack of Explicit Contribution**

The manuscript compares with literature but does not clearly state:

- What is new

For example:

Multiple comparisons are provided, but no statement such as
“This study adds…” or “This study is the first to…”

👉 Add a clear contribution statement.

**Overinterpretation**

The manuscript states:

*“could be explained by… unhealthy dietary and behavioral habits”*

👉 This implies causality, which is not supported by the cross-sectional design.

**Independent Associations (Incorrect Implication)**

The manuscript discusses factors such as:

- Diet
- NSAIDs
- Energy drinks

👉 However, these were not analyzed as **independent predictors in separate models**, so they should not be presented as independently associated.

**Implications vs Recommendations**

The section:

*“Implications and recommendations”*

mixes:

- Interpretation
- Actionable recommendations

👉 These should be **separated clearly**

**Recommendations Not Fully Supported**

Recommendations include:

*“pharmacological treatment… comprehensive mental health approaches”*

👉 These extend beyond:

- Variables measured in the study

**Residual Confounding Underemphasized**

The manuscript states:

*“variables such as academic stress or other mental health variables were not measured”*

👉 This is a **major limitation**, but it is not strongly emphasized in the discussion.

**Dose–Response Overstatement**

The manuscript states:

*“strengthens the plausibility of the association”*

👉 In cross-sectional studies, this should be phrased more cautiously.

**Conclusion and Recommendations**

The conclusion is well aligned with the results and appropriately avoids causal language. However:

- Recommendations are not clearly linked to findings
- They are not presented alongside the conclusion in a structured way

👉 Ensure:

- Recommendations are **directly derived from findings**
- They are clearly integrated with the conclusion

**🔑 Final Recommendation**

The manuscript addresses an important topic and demonstrates several strengths, including appropriate tools and analysis. However, revisions are required to improve:

- **Clarity of contribution to knowledge**
- **Handling of confounding (especially mental health and medications)**
- **Statistical reporting (effect sizes in Table 2)**
- **Separation of results vs interpretation**
- **Alignment between findings and recommendations**

Addressing these issues will substantially strengthen the manuscript and improve its suitability for publication.

If you want next, I can:

- Provide a **“Major revision decision letter” version**
- Or **rewrite specific weak sections (Discussion/Results) exactly as authors should correct them**

rewrite this providing more specific examples from the manuscript as describedin pasted text some comments are general and authors may not pinpoint the refered text in their manuscript. especialy for methods resuts and discuassion

Here is a **more precise, line-linked reviewer report**, with **highly specific references to the manuscript text**, especially strengthening the **Methods, Results, and Discussion critiques** so authors can directly identify what to revise.

**🧾 Peer Reviewer Report (Highly Specific, Text-Referenced Version)**

**Overall Assessment**

This manuscript investigates the association between GERD symptoms and sleep quality among medical students and addresses a relevant topic. The study is generally well organized and uses appropriate tools (PSQI, FSSG) and analysis (Poisson regression). However, several **methodological, analytical, and interpretational issues** remain. Many sections contain **general statements that are not sufficiently supported by the methods or results**, and some inconsistencies between sections require clarification.

**Title**

The title:

*“Association between gastroesophageal reflux symptoms and sleep quality…: a cross-sectional study”*

contains mild redundancy, as both “association between” and “cross-sectional study” signal a non-causal relationship. While acceptable, it can be tightened by removing “association between” without losing meaning.

**Keywords**

If the keyword list includes methodological terms such as “cross-sectional study,” these should be reconsidered. Keywords should reflect **content (GERD, sleep quality, medical students, Peru)** rather than study design, which is already specified in the title and methods.

**Abstract**

The abstract reports results but does not clearly define the **knowledge gap**. For example, while it likely states prevalence and associations, it does not explicitly clarify:

- What is unknown in **Latin American or Peruvian medical student populations**
- What distinguishes this study from prior work cited later (e.g., Iranian or Indian studies in the discussion)

👉 The abstract should explicitly state the **gap and contribution**, not just findings.

**🔴 Methods (Major Issues with Specific Examples)**

**1. Study Setting – insufficient detail**

The manuscript states:

*“medical students from a private university (Universidad Peruana Unión) in Lima, Peru”*

However, it does not describe:

- Whether this is a large or small institution
- Distribution across academic years
- Whether students are predominantly urban/residential

👉 This limits interpretation of findings such as:

high prevalence of GERD (76.6%)

**2. Sample size calculation – incomplete reporting**

The manuscript states:

*“resulting in a minimum required sample size of 160 participants”*

But does not report:

- Allowable error (e.g., 0.05)
- Formula used
- Whether finite population correction (N=698) was applied
- Whether non-response was accounted for

👉 Without these, the calculation **cannot be verified**

**3. Confounders – critical omissions**

The variables included are:

*“NSAIDs, energy drinks, coffee, alcohol, tobacco…”*

However, the discussion later states:

*“high prevalence of depression and anxiety”*
*“academic stress…”*

👉 These variables:

- Were **not measured**
- Were **not adjusted for**

This is a major inconsistency between **methods and discussion**, introducing **residual confounding**

Additionally, **medications affecting sleep** (e.g., sedatives, antidepressants) were not included, despite their strong relevance.

**4. Multicollinearity – incorrect handling**

The manuscript states:

*“age was categorized into tertiles to avoid this issue”*

👉 This is conceptually incorrect:

- Multicollinearity occurs between variables, not within a single variable
- Categorizing age does not resolve it

**5. Data quality control – not described**

Despite stating:

*“online survey… distributed via Microsoft Forms and WhatsApp”*

There is no mention of:

- Duplicate response control
- Completion checks
- Missing data handling

👉 This is a critical omission for online data collection.

**🔴 Results (Major Issues with Specific Examples)**

**1. Over-reliance on p-values without effect size**

The manuscript reports:

*“prevalence was significantly higher among students who reported NSAID consumption (93.5%, p=0.016)”*

and:

*“energy drink consumption (94.3%, p=0.012)”*

👉 These statements:

- Emphasize **statistical significance only**
- Do not provide **magnitude of association**

✔ Table 2 should include:

- **Crude prevalence ratios (cPR) with 95% CI**

**2. Table 2 – incomplete statistical reporting**

Table 2 includes:

- Frequencies (%)
- p-values

But lacks:

- Effect estimates (cPR)

Additionally, the legend states:

*“p value calculated using Chi-square test, Fisher’s exact test, or Mann–Whitney U test”*

👉 But does not specify:

- Which test corresponds to which variable

**3. Misinterpretation of statistical tests**

Chi-square results are used to imply associations, e.g.:

*“prevalence was significantly higher…”*

👉 However:

- Chi-square shows **presence of association only**
- Not direction or strength

**4. Interpretation within Results section**

The manuscript states:

*“a linear and directly proportional relationship was observed…”*

👉 This is **interpretation**, not pure reporting

✔ Should be replaced with:

“a positive association was observed”

**5. Extremely high prevalence not contextualized**

Reported:

- GERD = **76.6%**
- Poor sleep = **84.8%**

👉 These are unusually high, yet no caution is provided in Results

**6. Continuous vs categorical GERD – not justified**

The manuscript reports:

*“aPR: 1.27…” (categorical GERD)*
*“aPR: 1.010…” (continuous score)*

👉 No explanation is provided for:

- Why both approaches are used
- How they complement each other

**🔴 Discussion (Major Issues with Specific Examples)**

**1. No explicit contribution to knowledge**

The manuscript states:

*“previous studies… reported…”*
*“this prevalence exceeds global estimates…”*

👉 However, nowhere does it clearly state:

- “This study adds…”
- “This is the first study…”

👉 Contribution is implied but **not explicitly stated**

**2. Overinterpretation of findings**

The manuscript states:

*“could be explained by unhealthy dietary and behavioral habits…”*

👉 This implies causality, yet:

- Study is cross-sectional

Similarly:

*“factors that disrupt circadian rhythms…”*

👉 These are **mechanistic explanations not directly measured**

**3. Independent associations incorrectly implied**

The manuscript discusses:

- NSAIDs
- Energy drinks
- Spicy foods

👉 However:

- These were not modeled as **independent predictors of GERD or sleep**

**4. Inconsistency: variables discussed but not measured**

The manuscript states:

*“academic stress… depression and anxiety…”*

👉 But:

- These were **not included in the methods or models**

👉 This weakens validity of interpretation

**5. Implications and recommendations mixed**

The section:

*“Implications and recommendations”*

Includes:

- Interpretation
- Interventions (e.g., pharmacological treatment, diet modification)

👉 These should be **separated**

**6. Recommendations not fully derived from findings**

The manuscript suggests:

*“pharmacological treatment… mental health interventions…”*

👉 Yet:

- Mental health variables were **not measured**
- Medication use (sleep-related) not assessed

**7. Residual confounding underemphasized**

The manuscript states:

*“mental health variables were not measured…”*

👉 This is a **major limitation**, but treated as minor

**8. Dose–response overstatement**

The manuscript states:

*“strengthens the plausibility of the association”*

👉 Should be more cautious:

- Cross-sectional dose-response ≠ causal inference

**Conclusion and Recommendations**

The conclusion is appropriate and consistent with results:

*“GERD was significantly associated with…”*

However:

- Recommendations are not tightly linked to findings
- They extend beyond measured variables

👉 Recommendations should:

- Be **directly derived from results**
- Be presented alongside conclusion clearly

**🔑 Final Recommendation**

This manuscript is methodologically promising but requires **major revisions**, particularly in:

- Confounder handling (mental health, medications)
- Sample size transparency
- Statistical reporting (include cPR in Table 2)
- Removal of interpretation from Results
- Clear articulation of contribution
- Alignment between findings and recommendations

If you want next, I can:

- Convert this into a **“major revision decision letter” (editor style)**
- Or **rewrite the Results and Discussion exactly how authors should correct them**
