# Supplementary material for: Gastroesophageal reflux symptoms and sleep quality among medical students at a private university in Lima, Peru: A cross-sectional study
Source: PLoS One. 2026 Jun 1;21(6):e0348891. doi: 10.1371/journal.pone.0348891 (PMC13225408; doi:10.1371/journal.pone.0348891)
Supplement: Attachment — Submitted filename: Response_to_Reviewers.docx [file pone.0348891.s005.docx]

**RESPONSE TO REVIEWERS**

**Manuscript title:** Association between gastroesophageal reflux symptoms and sleep quality among medical students at a private university in Lima, Peru: a cross-sectional study

We sincerely thank the Academic Editor and all four Reviewers for their thorough, constructive, and detailed evaluation of our manuscript. Their comments have led to substantial improvements in the clarity, methodological transparency, and scientific rigor of the revised version. We have addressed every comment carefully, point by point, in the sections below. Changes made to the manuscript are described in each response, and the specific location of each change is noted.

## Reviewer 1

**Comment 1.1**."In the methods section, consider presenting the sample size calculation first, then the recruitment approach, and finally how incomplete records were handled. The study does not report the number of students approached or the number who declined participation."

We thank the reviewer for this organizational suggestion. We have reorganized the "Design, population, and sample" subsection so that the sample size calculation is presented first, followed by the sampling and recruitment approach, and finally the handling of incomplete data. However, due to convenience sampling we do not have the number of students invited to participate.

Modified text in Methods: “*A sample size calculation was performed considering a population of 698 students, a proportion of poor sleep quality of 83.9% [17], and a 95% confidence level, resulting in a minimum required sample size of 160 participants. Non-probabilistic convenience sampling was used*.”

**Comment 1.2.** "The definition of poor sleep quality requires clarification. The manuscript classifies poor sleep as a PSQI score ≥5; however, the validated cutoff defines poor sleep quality as a global PSQI score >5, with scores ≤5 indicating good sleep quality. The authors should clarify/justify the use of this alternative threshold."

We thank Reviewer 1 for this important methodological observation. The original validation study by Buysse et al. (1989) mentions “A post hoc cutoff score of 5 correctly identified 88.5% (131/ 148) of all patients and controls (kappa = 0.75, p < 0.001)”. Also, in two Peruvian validation studies (Ravelo Bobadilla, 2022; and Luna-Solis, 2015) a cutoff of ≥5 was applied. We have added these references.

**Comment 1.3**. "The link between multicollinearity assessment (VIF) and the decision to categorize age into tertiles requires clarification. VIF is used to detect collinearity among predictors. Please clarify the methodological basis for this decision."

We appreciate this observation. We acknowledge that the original wording was imprecise.

Modified text in Methods: “*Multicollinearity was assessed using the variance inflation factor (VIF). We observed that age, when included as a continuous variable, showed a high degree of multicollinearity. To reduce this collinearity while retaining age as a covariate in the adjusted model, age was categorized into tertiles*.”

**Comment 1.4**. "Given the findings of reference 14, the incremental contribution of this study is not immediately clear. The discussion should explicitly articulate how this study advances existing knowledge, including any novel population-specific insights or methodological contributions."

We thank reviewer for this important point. We have added a clear statement of the study's incremental contributions to the Discussion section.

Added paragraph in the Discussion section: “*Our study provides several incremental contributions to the existing literature on the relationship between GERD and sleep quality. First, it focuses on Peruvian medical students, a specific and understudied subgroup within the Latin American context. Second, unlike previous meta-analysis [14], which was based primarily on studies from the general population, our study analyzes a population with a distinct risk profile characterized by high academic stress, irregular dietary habits, and young age. Third, by using Poisson regression with robust variance, we obtained adjusted prevalence ratios that are more appropriate for cross-sectional, representing a methodological improvement over previous studies in this population that relied mainly on unadjusted comparisons. Finally, we identified a dose–response relationship between GERD symptom burden and poor sleep quality, which strengthens the biological plausibility of the observed association in this specific population*.”

## Reviewer 2

**Comment 2.1.** "Overall, check for grammatical flow and sentence formation of the entire manuscript, as some of the sentences are long, vague, and not up to the scientific standard."

We thank Reviewer 2 for this comment. We have carefully revised the entire manuscript for grammatical clarity, sentence structure, and scientific precision.

**Comment 2.2**. "Limit abbreviations in the abstract section."

We thank the Reviewer for this observation. We have revised the Abstract to reduce the use of abbreviations. Specifically, we removed the abbreviations for the Pittsburgh Sleep Quality Index and the Frequency Scale for the Symptoms of Gastroesophageal Reflux Disease. We retained GERD because it is used repeatedly throughout the Abstract.

**Comment 2.3 (Introduction).** "The authors didn't clearly describe what is already known regarding the association (and its degree) between sleep quality and GERD symptoms, particularly in the study population (medical students). Which gaps in the literature does the current study aim to narrow?"

We appreciate this important observation. We have expanded the Introduction to more explicitly describe: (1) what is known about the GERD–sleep quality association from the systematic review by Tan et al.; Reference 14; (2) what is known from the two prior cross-sectional studies in medical students (Teimouri & Amra, 2021; Sharma et al., 2018; References 15 and 16), highlighting their limitations — specifically, the absence of adjusted analyses; and (3) a clear statement of the knowledge gap: no study with adequate control for confounders has been conducted among Peruvian medical students, and context-specific evidence from this population is needed to guide local health interventions.

Added text in Introduction: “*A systematic review found that GERD is significantly associated with poorer sleep quality (OR: 1.47; 95% CI: 1.24 to 1.74) and shorter sleep duration (OR: 1.17; 95% CI: 1.12 to 1.21), although with some heterogeneity among studies [14]. Evidence in medical students is still scarce. A study in Iran reported an association between reflux symptoms and impaired sleep quality, while a study in India found that inadequate sleep and sleep-related habits were associated with GERD symptoms; however, both studies were cross-sectional and lacked adequate adjusted analyses to control for confounding [15,16]. This is particularly important in medical students, a population with a high frequency of poor sleep quality, irregular eating habits, and heavy academic workload, factors that may modify this relationship. To date, no study with adequate control for confounding has evaluated this association among Peruvian medical students. Therefore, context-specific evidence is needed to better characterize this relationship and support local preventive strategies*.”

**Comment 2.4 (Methods)** "The study period should be stated (e.g., from October 27 to November 28, 2024)."

We thank Reviewer for this comment. The study period (October 27 to November 28, 2024) was already stated in the Procedures subsection of the Methods. To improve visibility and completeness, we have also added the study period to the first paragraph of the Methods section ("Design, population, and sample").

Modified text in Methods: “*An analytical cross-sectional study was conducted among medical students from a private university (Universidad Peruana Unión) in Lima, Peru, between October 27 and November 28, 2024*.”

**Comment 2.5 (Methods).** "Inclusion and exclusion criteria should be clearly delineated and justified. Given that the study population is medical students (who are most likely more than or somewhere around 18 years old), what is the rationale for using the 18-year-old age cutoff for inclusion? Were the exclusion criteria limited to participants with incomplete data? Please mention if any additional exclusion was used. What defines incomplete data in the study?"

We thank Reviewer for these important questions. We have revised the Methods section to clearly separate and describe the inclusion and exclusion criteria. The inclusion criteria were medical students enrolled at Universidad Peruana Unión, aged ≥18 years, who voluntarily provided informed consent to participate. The exclusion criterion was the presence of at least one missing value in the study variables collected through the survey; however, no participants were excluded for missing data in the final dataset. Regarding the minimum age requirement of 18 years, although most medical students are adults, some first-year students may be younger than 18 years. This cutoff was established to ensure that all participants had full legal capacity to provide informed consent under Peruvian regulations. No additional exclusion criteria were applied.

**Comment 2.6 (Methods)** "In participant selection, why do you prefer to include only students attending an in-person academic event? This can introduce a potential selection bias because students with frequent GERD symptoms and poor sleep may have increased absenteeism, which could lead to their exclusion and underrepresentation in the study."

We acknowledge that recruiting participants during an in-person academic event could raise concerns about potential selection bias if students with more severe GERD symptoms or poorer sleep quality were less likely to attend. However, this event is held annually by the medical school, includes students from all academic years, and is usually mandatory as part of academic activities contributing to course assessment. Therefore, the likelihood of substantial underrepresentation due to absenteeism is likely low. In addition, to broaden participation beyond those present at the event, we also disseminated the survey link via WhatsApp during the data collection period.

Modified text in Methods, Procedures: “*The survey was distributed during an in-person academic event organized by the medical school, which included students from all academic years. This event is usually mandatory and forms part of the academic evaluation, which likely reduced the possibility of substantial selection bias due to absenteeism*.”

**Comment 2.7 (Methods).** "Why did you prefer to use non-probabilistic convenience sampling? Even though non-probabilistic convenience sampling can be useful for pilot studies or exploratory research, in analytical studies like this, it can pose a significant limitation. The authors should clarify these points and acknowledge them as limitations if necessary."

We appreciate this comment. Non-probabilistic convenience sampling was adopted due to logistical constraints, as no official list of enrolled students with contact information was available to enable probability-based sampling. We acknowledge that this approach limits the external generalizability of our findings and may introduce selection bias. We have added a brief justification of this methodological choice in the Methods section.

Modified text in Methods: “*Non-probabilistic convenience sampling was used because no official student list was available to perform random sampling*.”

**Comment 2.8 (Methods).** "Was block sampling by academic year used to affirm the balanced distribution of participants across academic years?"

We thank the reviewer for this question. Block sampling by academic year was not used. For the same reason described above, we did not have access to an official student list that would allow random selection within academic-year strata. Therefore, non-probabilistic convenience sampling was used. Nevertheless, the survey was distributed during an academic event that included students from all academic years, which facilitated the participation of students across the different stages of training.

**Comment 2.9 (Methods).** "Was sample size calculation for detecting associations performed?"

We thank Reviewer for raising this point. A formal statistical power calculation for detecting a specific magnitude of association was not performed, as no prior reliable estimate of the association between GERD and sleep quality in this specific population was available at the time of study planning. We have added this clarification to the Methods section.

Modified text in Methods: “*A sample size calculation specifically for detecting an association was not performed because no preliminary evidence was available in this population using the same instruments to assess both GERD symptoms and sleep quality. Therefore, the sample size was estimated using a descriptive approach, considering a population of 698 students, a proportion of poor sleep quality of 83.9% [17], and a 95% confidence level, resulting in a minimum required sample size of 160 participants*.”

**Comment 2.10 (Methods).** "The dependent variable in the study was sleep quality, specifically "during the last month". Do you use a similar time frame for the independent variable (GERD symptoms)? This is critical to ensure a temporal association between GERD symptoms and sleep quality."

We thank Reviewer 2 for this critical observation. The PSQI assesses sleep quality over the past month. The FSSG, as originally developed by Kusano et al. (2004; Reference 19), does not specify an explicit recall period; response options reflect habitual symptom frequency ("never," "occasionally," "sometimes," "often," "always") without a defined time window. We acknowledge that this difference in temporal framing between the two instruments is a methodological limitation, as the GERD symptom burden captured by the FSSG may not correspond precisely to the same one-month period assessed by the PSQI. We have added this as an explicit limitation in the revised manuscript.

Added text in Discussion, Limitations: “*Furthermore, the PSQI assesses sleep quality during the last month, whereas the FSSG does not specify an explicit recall period, which may have introduced temporal misalignment between the measurement of the exposure and the outcome*.”

**Comment 2.11 (Methods).** "Some relevant confounders based on prior literature seem to be missing. This includes use of symptomatic treatments for GERD, like PPIs; comorbidities, including psychiatric illness and other medical conditions known to impact sleep quality; self-reported depression and anxiety symptoms; physical activity."

We acknowledge that some potentially relevant variables, such as physical activity and mental health-related factors, may act as confounders in the association between GERD symptoms and sleep quality and were not measured in this study. In contrast, the use of medication for GERD was not considered a confounder, since it is more likely to affect GERD symptom burden directly rather than sleep quality independently. Nevertheless, we recognize that the absence of some unmeasured variables may have introduced residual confounding, and this has been explicitly acknowledged in the Limitations section: “*Another important factor is that variables such as academic stress or other mental health variables were not measured, which could have resulted in residual confounding*.”

**Comment 2.12 (Methods).** "In describing the statistical analysis, clearly justify your model choice, including why robust variance was used for technical clarity, and report the confidence interval."

We thank the reviewer for this suggestion. We have revised the Statistical Analysis paragraph to more clearly justify the use of Poisson regression with robust variance.

Added text in Methods: “*This regression approach was used given the binary nature of the outcome. Robust (sandwich) variance estimators were applied to obtain valid standard errors when Poisson models are fitted to binary data*. “

**Comment 2.13 (Methods)** "Authors should clearly state how confounders were selected. Mention whether clinical relevance, statistical criteria, or a combination of both was used."

We appreciate this observation. We have clarified in the revised Methods section that confounders were selected a priori based on clinical relevance and biological plausibility, as supported by prior literature on GERD and sleep quality, and were not selected based on statistical significance (p < 0.2) in the bivariate analysis. All sociodemographic and lifestyle variables considered to be plausible confounders of the GERD–sleep quality association were included in the adjusted model regardless of their bivariate p-value.

Revised text in Methods: “*For inclusion of variables in the adjusted model, we followed an epidemiological approach by including all potential confounders of the association based on clinical relevance, biological plausibility, and prior literature, rather than on statistical significance in the bivariate analysis. Accordingly, all sociodemographic and lifestyle variables considered plausible confounders of the association were included in the adjusted model regardless of their bivariate p-values [23,24]*.”

**Comment 2.14 (Results).** ""Initially, 176 students were surveyed, of whom 5 did not meet the inclusion criteria; therefore, 171 participants were finally analyzed." Why were students who don't meet the inclusion criteria surveyed in the first place? Modify the description accordingly."

After data collection, five respondents were excluded because they were younger than 18 years and therefore did not meet the predefined inclusion criteria. We have revised the Results section to clarify this point.

Modified text in Results: “*Initially, 176 students were surveyed, of whom 5 participants aged under 18 years were excluded; therefore, 171 participants were finally analyzed (Fig 1).”*

**Comment 2.15 (Results).** "It would be better and more informative if participant characteristics were reported categorized for students with GERD and without GERD to account for group differences."

We thank the reviewer for this suggestion. In the present study, the main dependent variable of interest was sleep quality; therefore, participant characteristics were presented according to sleep quality categories. This approach was aligned with the primary objective of the study, which was to evaluate the association between GERD symptoms and sleep quality, rather than to identify factors associated with GERD itself. For this reason, we considered the current presentation of participant characteristics according to the outcome of interest to be the most appropriate.

**Comment 2.16 (Discussion).** "The alarmingly high prevalence of GERD compared with previous studies needs better discussion. Put the potential reasons for the previous studies' higher, lower, and similar findings compared to your study separately."

We thank the reviewer for this important observation. We have substantially revised the Discussion to provide a more structured comparison of our observed GERD prevalence (76.6%) with previous studies and additional potential explanations for the higher prevalence in our study, including.

Modified text in Discussion “*Previous studies among Peruvian university students have reported similar or even lower prevalences of GERD. A thesis conducted among medical students at another Peruvian university reported a GERD prevalence of 75%, while another study conducted in medical students in Huacho, Peru, reported a lower prevalence of 42% [22,25]. However, the frequency observed in our study greatly exceeds the global estimate (14%) and that reported for Latin America (12.9%) [9]. Several factors may explain these differences. The use of the FSSG captures the frequency of reflux-related symptoms rather than a clinically confirmed diagnosis, which may yield higher prevalence estimates. Also, convenience sampling may have favored the participation of students who were more health-conscious or more likely to report symptoms. Furthermore, the high frequency of unhealthy dietary and behavioral habits identified in the sample, such as excessive consumption of coffee and energy drinks, use of NSAIDs, and intake of spicy foods, may have contributed to the elevated prevalence observed [26,27]. Additional context-specific factors that could explain the elevated GERD prevalence include academic stress, sleep deprivation, irregular eating schedules, and greater awareness and reporting of symptoms due to medical training.”*

**Comment 2.17 (Discussion).** "We don't know whether the mentioned behaviors are more common in those students with or without GERD. Reporting participant characteristics categorized for students with GERD and without GERD might be beneficial."

We thank the reviewer for this suggestion. The mention of these behaviors in the Discussion was based on prior literature, particularly Reference 27, which is a global systematic review of risk factors for GERD. We considered that this source provides broader and more robust evidence on behavioral factors associated with GERD than the descriptive comparison that could be derived from our study. In addition, the main objective of our study was not to identify factors associated with GERD, but to evaluate the association between GERD symptoms and sleep quality. For this reason, participant characteristics were presented according to the dependent variable of interest, namely sleep quality, rather than according to GERD status.

**Comment 2.18 (Discussion)** "Some of the factors mentioned (e.g., depression and anxiety, frequent use of electronic devices at night) could have been measured in the study, which would also have mitigated the confounding factors in assessing the association between GERD and poor sleep."

We thank Reviewer 2 for this important observation. We agree that depression, anxiety, and electronic device use, factors mentioned in the Discussion as potential contributors to poor sleep quality. These factors have been repositioned in the Limitations section as potential sources of residual confounding. Text in limitations: “*Another important factor is that variables such as academic stress or other mental health variables were not measured, which could have resulted in residual confounding*.”

**Comment 2.19 (Discussion).** "In the final paragraph, the authors have focused on describing findings from previous studies rather than discussing findings from the current study and analytically comparing it with previous reports. The whole paragraph needs to be modified accordingly."

We appreciate this important structural comment. We have rewritten the paragraph providing a comparison with prior studies: our direction of association is consistent with the meta-analysis by Tan et al. (OR: 1.47; Reference 14) and the unadjusted findings of Teimouri & Amra (2021; Reference 16), while contrasting with the null finding of Sharma et al. (2018; Reference 15), for which we offer potential methodological explanations.

Modified text in Methods: “*Regarding the association between GERD and sleep quality, a previous systematic review including 22 case–control or cohort studies found that GERD was associated with higher odds of poor sleep quality (OR: 1.47; 95% CI: 1.21 to 1.79) and shorter sleep duration (OR: 1.17; 95% CI: 1.12 to 1.21) [14]. Similarly, a previous cross-sectional study conducted in Iran found that medical students with GERD symptoms had higher frequencies of poor sleep quality (p<0.001); however, adjusted regression analyses to control for confounding were not performed [16]. Another cross-sectional study conducted among medical students in India found that inadequate sleep was not associated with the presence of GERD (p=0.260); however, going to sleep within one hour after dinner was associated with a higher frequency of GERD (p=0.001) [15]. Our findings are consistent with the systematic review and the Iranian study, as we also found that GERD was associated with a higher prevalence of poor sleep quality, but differ from the Indian study, which did not observe this association, probably due to methodological differences such as the lack of adjusted analyses and differences in the sleep-related measures used. Moreover, in our study we observed a dose–response relationship between greater GERD symptomatology and a higher prevalence of poor sleep quality, which strengthens the plausibility of the association. From a biological plausibility perspective, nocturnal reflux may induce micro-arousals and sleep fragmentation due to nocturnal acid exposure and autonomic alterations that reduce the parasympathetic activity typical of sleep [31–33]. However, given the cross-sectional design, temporality cannot be established, and the relationship may be bidirectional, as suggested by previous evidence [14].*”

## Reviewer 3

**Comment 3.1**. “The FSSG cut-off of >8 was validated in a Japanese clinical population, not in Peruvian university students; authors must either cite a validated adaptation or reframe results as "GERD symptom burden" rather than clinical GERD prevalence."

We thank Reviewer 3 for this critical methodological observation. We fully acknowledge that the FSSG was originally developed and validated in a Japanese clinical patient population (Kusano et al., 2004; Reference 19) and that no formal cross-cultural validation study in Peruvian university students has been published to date. However, the instrument has been used in this population in prior Peruvian studies (References 20 and 21), which provided the precedent for its application. In response to this comment, throughout the manuscript, we have replaced the term GERD prevalence by GERD symptom burden.

**Comment 3.2**. "A 1% prevalence increase per one-point FSSG increment is statistically significant but clinically modest; the authors should illustrate the magnitude by comparing predicted prevalence between a student at the GERD threshold (score 8) and one with moderate-severe burden (score ~30)."

We thank the reviewer for this thoughtful suggestion. We agree that clinical interpretability of the continuous FSSG estimate is important. However, we decided not to include a comparison between an FSSG score of 8 and a score of 30 because these cut points are somewhat arbitrary and were not prespecified in our analysis plan. In addition, focusing on two selected values could oversimplify the interpretation of a continuous association across the full range of symptom burden. To address interpretability, we retained the post-estimation figure, which graphically presents the relationship between the GERD symptom score and the adjusted prevalence of poor sleep quality across the observed range of values, providing a more complete representation of the association.

**Comment 3.3**. "The reliance on convenience sampling and self-reported data introduces potential selection and reporting biases, which should be more explicitly acknowledged and discussed in terms of their impact on internal and external validity."

We thank Reviewer 3 for this observation. We have substantially expanded the Limitations section to address these concerns explicitly. Regarding external validity: convenience sampling of students attending an in-person event and recruited via WhatsApp likely resulted in overrepresentation of more health-conscious and engaged students, which may have inflated the observed prevalences of both GERD symptoms (76.6%) and poor sleep quality (84.8%). Regarding internal validity: the self-reported nature of both outcome measures introduces the risk of recall bias (particularly for the PSQI, which requires recalling sleep patterns over the past month) and social desirability effects.

Modified text in Discussion, Limitations: “*Some limitations should be considered. The cross-sectional design prevents establishing causality and directionality of the association. Convenience sampling may limit external validity and generate selection bias, as recruitment during an in-person academic event and through WhatsApp may have favored the participation of more health-conscious or more engaged students, potentially leading to overestimation of the prevalences of both GERD symptoms and poor sleep quality Self-reported nature of the data may have affected internal validity through reporting biases, including recall bias and social desirability bias.”*

## Reviewer 4

**Major Comment 4.1.** "The manuscript does not clearly articulate what is unknown in the specific context of Peruvian or Latin American medical students. Action required: Explicitly state the knowledge gap; clearly define how this study adds to existing literature."

We appreciate this important observation. We have expanded the Introduction to more explicitly describe: (1) what is known about the GERD–sleep quality association from the systematic review by Tan et al.; Reference 14; (2) what is known from the two prior cross-sectional studies in medical students (Teimouri & Amra, 2021; Sharma et al., 2018; References 15 and 16), highlighting their limitations — specifically, the absence of adjusted analyses; and (3) a clear statement of the knowledge gap: no study with adequate control for confounders has been conducted among Peruvian medical students, and context-specific evidence from this population is needed to guide local health interventions.

Added text in Introduction: “*A systematic review found that GERD is significantly associated with poorer sleep quality (OR: 1.47; 95% CI: 1.24 to 1.74) and shorter sleep duration (OR: 1.17; 95% CI: 1.12 to 1.21), although with some heterogeneity among studies [14]. Evidence in medical students is still scarce. A study in Iran reported an association between reflux symptoms and impaired sleep quality, while a study in India found that inadequate sleep and sleep-related habits were associated with GERD symptoms; however, both studies were cross-sectional and lacked adequate adjusted analyses to control for confounding [15,16]. This is particularly important in medical students, a population with a high frequency of poor sleep quality, irregular eating habits, and heavy academic workload, factors that may modify this relationship. To date, no study with adequate control for confounding has evaluated this association among Peruvian medical students. Therefore, context-specific evidence is needed to better characterize this relationship and support local preventive strategies*.”

**Major Comment 4.2.** "The study setting is described only as "a private university (Universidad Peruana Unión) in Lima, Peru." This lacks sufficient contextual detail. Action required: Describe the institution (type, size, structure of medical program, student distribution)."

We appreciate this suggestion. We have expanded the study setting description in the Methods section.

Added text in Methods: “*An analytical cross-sectional study was conducted among medical students from a private university (Universidad Peruana Unión) in Lima, Peru, during between October 27 and November 28, 2024. This university has a campus in Lima where the study was carried out within the Faculty of Health Sciences, specifically in the School of Medicine. The medical program is a face-to-face undergraduate program with a duration of seven academic years, structured into three training stages: basic sciences, clinical sciences, and internship*.”

**Major Comment 4.3.** "The manuscript reports a minimum required sample size of 160 participants but omits: allowable error (precision), formula used, finite population correction, non-response adjustment."

We thank the reviewer for this observation. We have added the required details about sample size calculation.

Modified text in Methods: “*Therefore, the sample size was estimated using a descriptive approach in Epidat V4.2 software, considering a finite population of 698 students, a proportion of poor sleep quality of 83.9% [17], a 95% confidence level, and a 5% margin of error, resulting in a minimum required sample size of 160 participants. Non-probabilistic convenience sampling was used because no official student list was available to perform random sampling*.”

**Major Comment 4.4.**"The manuscript includes lifestyle variables but omits mental health (stress, anxiety, depression) and medications affecting sleep. Yet later discusses "academic stress… depression and anxiety." Action required: Either include these variables in analysis (if available) or strongly emphasize as a major limitation."

We fully acknowledge this critical gap. Mental health variables and medications that affect sleep were not assessed in this study. Their absence introduces residual confounding, the direction of which is difficult to determine a priori. We do not have these variables in the database, hence is a limitation.

Added text in Discussion, Limitations: “Another important factor is that variables such as academic stress or other mental health variables were not measured, which could have resulted in residual confounding.”

**Major Comment 4.5.** "Age was categorized into tertiles to avoid multicollinearity." Action required: Revise this statement. Provide correct approach to assessing multicollinearity (e.g., VIF)."

This comment is addressed in conjunction with Reviewer 1, Comment 1.3 above. We acknowledge that the original wording was imprecise.

Modified text in Methods: “*Multicollinearity was assessed using the variance inflation factor (VIF). We observed that age, when included as a continuous variable, showed a high degree of multicollinearity. To reduce this collinearity while retaining age as a covariate in the adjusted model, age was categorized into tertiles*.”

**Major Comment 4.6.** "Data were collected via Microsoft Forms and WhatsApp, but no mention of: duplicate checks, missing data handling, validation procedures. Action required: Describe data quality assurance measures."

We thank Reviewer 4 for this important observation. We have clarified the data quality assurance procedures in the revised manuscript. During data cleaning, we verified that there were no duplicate records, no missing values, and no implausible data entries in the dataset. Because none of these issues were identified, no participants were excluded due to incomplete data.

Added text in Methods, Statistical Analysis: “*Data from the online survey were exported to Microsoft Excel, where data cleaning and coding were performed. During this process, the database was reviewed to identify duplicate records, missing values, and implausible entries, but no such issues were found*.”

**Major Comment 4.7.** "Over-reliance on p-values. No effect size in Table 2. Action required: Include crude prevalence ratios (cPR) with 95% CI in Table 2. Reduce emphasis on p-values alone."

We agree that effect size measures are essential for the main association analyses, and for this reason we reported crude and adjusted prevalence ratios with 95% confidence intervals in Table 3, which directly addresses the primary objective of the study. However, the purpose of Table 2 was descriptive and exploratory, to present participant characteristics according to the dependent variable of interest. The objective of this study was not to identify factors associated with sleep quality. Therefore, we chose not to calculate prevalence ratios for variables in Table 2, as such estimates could be misinterpreted as etiologic effects despite arising from bivariate comparisons alone, which may be affected by the “Table 2 fallacy.” For this reason, we retained p-values in Table 2 only as descriptive indicators of group differences and focused the interpretation of effect sizes on the regression models presented in Table 3.

**Major Comment 4.8.** "Table 2 legend does not specify which test was used for each variable. Table 1 does not clearly separate counts and percentages."

We appreciate these formatting observations. We have revised the footnote of Table 2 to specify which statistical test was applied to each variable type.

Added footnotes in Table 2:

a. Chi-square test.

b. Mann–Whitney U test.

c. Fisher’s exact test.

Added footnotes in Table 1:

* Absolute frequencies (n) and relative frequencies (%)

**Major Comment 4.9.** "A linear and directly proportional relationship was observed…" — Remove interpretation from Results section. Replace with neutral wording."

We thank Reviewer 4 for this precision comment.

Modified text in Results: “*Graphically, a positive association was observed between increasing GERD symptom score and the adjusted prevalence of poor sleep quality (Fig 2).”*

**Major Comment 4.10.** "Could be explained by unhealthy dietary and behavioral habits…" — Avoid overinterpretation. Use cautious language ("may be associated with")."

We thank the reviewer for this important consideration. We have carefully reviewed the entire Discussion for causal or deterministic language and replaced all such instances with appropriately cautious alternatives.

Modified text in Discussion: “*Furthermore, the high frequency of unhealthy dietary and behavioral habits identified in the sample, such as excessive consumption of coffee and energy drinks, use of NSAIDs, and intake of spicy foods, may have contributed to the elevated prevalence observed [26,27]*.”

**Major Comment 4.11.** "The manuscript compares findings but does not explicitly state what this study adds. Action required: Add a clear statement of contribution."

We have added a dedicated paragraph in the Discussion explicitly stating the study's contributions.

Added text in Discussion: “*Our study provides several incremental contributions to the existing literature on the relationship between GERD and sleep quality. First, it focuses on Peruvian medical students, a specific and understudied subgroup within the Latin American context. Second, unlike previous meta-analysis [14], which was based primarily on studies from the general population, our study analyzes a population with a distinct risk profile characterized by high academic stress, irregular dietary habits, and young age. Third, by using Poisson regression with robust variance, we obtained adjusted prevalence ratios that are more appropriate for cross-sectional, representing a methodological improvement over previous studies in this population that relied mainly on unadjusted comparisons. Finally, we identified a dose–response relationship between GERD symptom burden and poor sleep quality, which strengthens the biological plausibility of the observed association in this specific population*.”

**Major Comment 4.12.** ""Implications and recommendations" mixes interpretation and action. Separate into: Implications / Recommendations."

We thank Reviewer 4 for this structural suggestion. We have divided the "Implications and recommendations" subsection into two clearly labeled subsections.

**Major Comment 4.13/4.14.** "Selection bias not sufficiently specified; online survey limitations not explicitly acknowledged; residual confounding underemphasized; omission of sleep medications; lack of local FSSG validation; reporting bias explanation needs strengthening."

We have comprehensively revised this section to address all six sub-points:

1. Selection bias, added text: “*Convenience sampling may limit external validity and generate selection bias, as recruitment during an in-person academic event and through WhatsApp may have favored the participation of more health-conscious or more engaged students, potentially leading to overestimation of the prevalences of both GERD symptoms and poor sleep quality*.”

2. Online survey limitations, added text: “*Self-reported nature of the data may have affected internal validity through reporting biases, including recall bias and social desirability bias*.”

3. Residual confounding bias, added text: “*Another important factor is that variables such as academic stress, mental health factors, and sleep medication use were not measured, which could have resulted in residual confounding and biased the observed association in either direction*.”

4. Medications affecting sleep, added text: “*Another important factor is that variables such as academic stress, mental health factors, and sleep medication use were not measured, which could have resulted in residual confounding and biased the observed association in either direction*.”

5. Lack of local FSSG validation, added text: “*In addition, FSSG and PSQI are screening instruments and do not correspond to a confirmed clinical diagnosis and although the FSSG has been used in Peruvian medical students, its psychometric properties have not been formally validated in this local population, which may affect the accuracy of GERD symptom classification*.”

6. Reporting bias, added text: “*Convenience sampling may limit external validity and generate selection bias****,*** *as recruitment during an in-person academic event and through WhatsApp may have favored the participation of more health-conscious or more engaged students, potentially leading to overestimation of the prevalences of both GERD symptoms and poor sleep quality* ***S****elf-reported nature of the data may have affected internal validity through reporting biases, including recall bias and social desirability bias*.”

**Major Comment 4.15.** ""Implementing systematic screening… and developing timely interventions…" — Too vague. Specify: screening tools, lifestyle interventions, clinical pathways."

We thank the reviewer for this suggestion.

Modified conclusion: “*These findings highlight the use of simple screening tools for GERD symptoms and sleep quality in university health settings and the implementation of targeted interventions focused on sleep hygiene* *and reduction of reflux-related behaviors. Students with persistent or more severe symptoms could also benefit from referral pathways for clinical evaluation and management*.”

**Minor Comment 4.1.** "Reduce redundancy ("association between" + "cross-sectional study")."

We have considered this suggestion carefully. Modified Title: “*Gastroesophageal reflux symptoms and sleep quality among medical students at a private university in Lima, Peru: a cross-sectional study*”

**Minor Comment 4.2**. "Remove study design from keywords. Focus on content terms."

We have revised the keyword list to remove "Cross-sectional studies".

**Minor Comment 4.3 Abstract.** "Add: knowledge gap, contribution, effect estimates (aPR with CI)."

We have revised the Abstract.

Knowledge gap added text in background: “*Introduction: Poor sleep quality is common among medical students and is associated with academic and health-related consequences. Gastroesophageal reflux disease (GERD) may contribute to sleep disturbances through biological and behavioral mechanisms, however, evidence in medical students is limited and previous studies have not adequately controlled for confounding*.”

Contributions added text in conclusions: “*Conclusion: GERD symptoms were significantly associated with poor sleep quality among Peruvian medical students. This study provides context-specific adjusted evidence from an understudied Latin American medical student population. These findings support the need for screening strategies and early interventions targeting both conditions in this population*.”

aPR are already mentioned in the abstract: “*Results: A total of 171 students were analyzed (median age: 22 years; 64.9% female). The prevalence of GERD symptom burden was 76.6%, and poor sleep quality was observed in 84.8% of participants. Students with GERD symptom burden had a higher prevalence of poor sleep quality compared to those without GERD (aPR: 1.27; 95% CI: 1.03 to 1.58). Additionally, for each one-point increase in the GERD symptom score, the prevalence of poor sleep quality increased by 1.0% (aPR: 1.01; 95% CI: 1.003 to 1.018), demonstrating a dose–response relationship*.”

**Minor Comment 4.4.** "No mention of missing values or handling approach."

Added text in Methods, Statistical analysis: “*Data from the online survey were exported to Microsoft Excel, where data cleaning and coding were performed. During this process, the database was reviewed to identify duplicate records, missing values, and implausible entries, but no such issues were found*.”

**Minor Comment 4.5.** "GERD (76.6%) and poor sleep quality (84.8%) are very high. Briefly acknowledge possible reasons (selection bias, measurement)."

Added text in Discussion, limitations: “*Convenience sampling may limit external validity and generate selection bias****,*** *as recruitment during an in-person academic event and through WhatsApp may have favored the participation of more health-conscious or more engaged students, potentially leading to overestimation of the prevalences of both GERD symptoms and poor sleep quality*”.

**Minor Comment 4.6.** "Expand to include: appropriate analytical approach (PR instead of OR), use of standardized tools."

Added text in Methods, Statistical analysis: “*This regression approach was used given the high-prevalence binary nature of the outcome. Robust (sandwich) variance estimators were applied to obtain valid standard errors when Poisson models are fitted to binary data*.”

**Minor Comment 4.7.** "Add risk of misclassification bias. Improve balance between strengths and limitations."

Added text in Discussion, limitations: “*This may have introduced non-differential misclassification of the exposure, potentially biasing the observed association toward the null*.”

Added text in Discussion, strengths: “*The use of both dichotomous and continuous GERD measures also allowed a more comprehensive assessment of the association, including a dose–response pattern*.”
